# The Botanical Labours of Mutis

**Published:** 1843-09

**Authors:** 


*The Botanical Labours of Mutis.*—A sketch of the labours of the extraordinary botanist of New Grenada, termed by Linnæus, "Phytologorum Americanorum Princeps," from the pen of Humboldt, translated by Colonel Wright, late governor of the province of Loxa, was read. It was Mutis who first mado known the true characters of the genus Cinchona, and who discovered the quinquina tree in the woods of Tena, near Santa Fé de Bogota, and also in the route from Honda to Villeta, and at the Mesa de Chinga. The cinchona tree is now to be found on the whole length of the Cordilleras, at an elevation of from seven hundred to fifteen hundred toises, upon an extent of about six hundred leagues from La Paez and Chuquisaca, to the mountains of Santa Martha and Merida. Mutis has the merit of being the first to distinguish the different species of cinchona, some of which, with downy petals, are much more active than others whose surface is smooth. He has proved that the active species, whose properties vary with the organic structure and form, should not be indiscriminately employed. Nor were the labours of Mutis confined to the genus Cinchona, most of the genera Alstonia, Vallea, Burmadesia, Escallonia, Manettia, Acæna, Brathys, Myroxylon, Befaria, Telipiogon, and many others, published in Linnæus's supplement, are due to the sagacity of the botanist of Santa Fé. Among the useful plants in medicine and commerce described by Mutis we must reckon the Phytrochia emetica, or ipecacuanha of the river Magdalena; the Toluifera and the Myroxylon, which yield the balsams of Tolu and Peru; the Winteria Granadensis, and the Alstenia theaformis, which latter furnishes the tea of Bogota, the infusion of which cannot be too strongly recommended to travellers who remain any length of time exposed to the rain of the tropics. To Mutis, also, is owing the discovery of the Mikania Guaco, the most powerful antidote to the bite of venomous serpents; it is of the family of the Compositæ, and is known in its native country by the name of the Vejuco del Guaco. Zoology, the atmospheric currents, and mathematics, also engage a portion of the attention of this extraordinary man. He never lost sight of the great problems of the *physique* of the universe. He travelled over the Cordilleras, barometer in hand. He determined the medium temperature of those table lands which appeared like islets in the middle of the ærial ocean. His taste for the physical sciences, and active curiosity directed to the development of the phenomena of organisation, and meteorology, maintained the empire of his mind to the last moment of his life. Of him, Linnæus truly said, *Nomen immortale quod nulla ætas unquam delebit.*

*London Lancet*, July 1, 1843.

